# Endodontic Misconceptions Among Dental Patients: Prevalence and Association with Tooth Extraction Preference

**DOI:** 10.3290/j.ohpd.c_2844

**Published:** 2026-10-21

**Authors:** Alaa Saud Aloufi, Hatim Abdulqader Qurban, Hosam Aabed Alofi, Mustafa Mohammed Alqazanli, Heba Saud Albalawi, Reem Husain Alhammad, Joud Mohamed Alotaibi

**Affiliations:** a Alaa Saud Aloufi Assistant Professor in Endodontics, Endodontic Division, Department of Restorative Dental Sciences, College of Dentistry, Taibah University, Madinah, Saudi Arabia. Study design, conducting the study, analysing the data, writing and reviewing the manuscript.; b Hatim Abdulqader Qurban Assistant Professor in Endodontics, Endodontic Division, Department of Restorative Dental Sciences, College of Dentistry, Taibah University, Madinah, Saudi Arabia. Study design, conducting the study, analysing the data, writing and reviewing the manuscript.; c Hosam Aabed Alofi Dental Intern, College of Dentistry, Taibah University, Madinah, Saudi Arabia. Study design, conducting the study, analysing the data, writing and reviewing the manuscript.; d Mustafa Mohammed Alqazanli Dental Intern, College of Dentistry, Taibah University, Madinah, Saudi Arabia. Study design, conducting the study, analysing the data, writing and reviewing the manuscript.; e Heba Saud Albalawi Dental Intern, College of Dentistry, Taibah University, Madinah, Saudi Arabia. Study design, conducting the study, analysing the data, writing and reviewing the manuscript.; f Reem Husain Alhammad Dental Intern, College of Dentistry, Taibah University, Madinah, Saudi Arabia. Study design, conducting the study, analysing the data, writing and reviewing the manuscript.; g Joud Mohamed Alotaibi Dental Undergraduate Student, College of Dentistry, Taibah University, Madinah, Saudi Arabia. Study design, conducting the study, analysing the data, writing and reviewing the manuscript.

**Keywords:** endodontics, myths, root canal therapy, tooth extraction

## Abstract

**Purpose:**

Part of proper oral health care is recognising and correcting dental myths and misconceptions that often discourage patients from seeking proper treatment decisions. Although general dental myths are well studied, little is known about the prevalence of specific endodontic misconceptions, especially those related to the effects of root canal treatment (RCT) on systemic health and their correlation with patients’ preference for extraction over RCT. This study aimed to investigate the prevalence of endodontic myths among adult dental patients and to explore potential associations with demographic variables and with a preference for tooth extraction over RCT.

**Methods and Materials:**

A self-administered paper questionnaire was distributed in person at 11 dental hospitals and private clinics in Almadinah Almunawarah, Kingdom of Saudi Arabia. Participants in the study included adults without cognitive impairments. The questionnaire collected sociodemographic data, history of previous RCT, agreement with 10 endodontic myths, and preference for extraction over RCT. To ensure understanding and clarity of the questionnaire, a pilot test was carried out. The data were then analysed using descriptive statistics, Chi-square, and multivariable logistic regression tests using the Statistical Package for the Social Sciences (SPSS) version 25.0. The *p* value was set at *p* ≤ 0.05.

**Results:**

A total of 463 participants completed the questionnaire. The most commonly endorsed myths were that root canal treatment can only be done in multiple visits (73.0%), that antibiotics are necessary during treatment (68.5%), and that root-canal-treated teeth become weak and brittle (63.1%). Almost a third of the patients (30.9%) believed that a root-canal-treated tooth remains infected; however, myths about systemic diseases were less common; these include beliefs that RCT causes cancer (6.3%), cardiovascular disease or heart attacks (10.8%), or chronic illnesses (14.3%). Older participants, lower educational levels and prior RCT experience showed significant endorsement of specific misconceptions (*p* < 0.05). Bivariate analyses revealed that several misconceptions were significantly associated with a greater preference for tooth extraction, specifically those related to systemic health. Patients who believed that root canal treatment (RCT) causes cancer, that RCT-treated teeth remain infected and that all root-canal-treated teeth should be extracted were more likely to prefer extraction than those who did not hold these beliefs (all *p* < 0.05).

**Conclusion:**

Endodontic myths were common among patients in Almadinah Almunawara. Misconceptions concerning systemic health effects, persistence of infection, and a perceived link between RCT and cancer were associated with a preference for tooth extraction over RCT. Age, educational level, and no previous RCT experience were associated with endorsement of specific misconceptions. Targeted public education and effective dentist-patient communication may help correct these misconceptions, improve acceptance of evidence-based endodontic care, and support preservation of natural dentition.

Proper oral healthcare comprises recognising and addressing common dental myths and misunderstandings that often lead patients to make incorrect decisions.^1^ Myths consist of beliefs, customs, and cultural identities that lack a verifiable foundation or scientific rationale.^15,25
^ These misconceptions have become deeply embedded in the lives of individuals across the globe. A major obstacle to dental and endodontic care is the false beliefs that discourage people from seeking oral health services.^12^


Endodontic therapy, commonly known as root canal treatment (RCT), is a tooth-preserving procedure used to manage pulpal and periradicular disease.^30^ Although the RCT success rate has improved in recent years, with around 95% in preserving a natural tooth, it continues to carry a strong negative image among many patients.^18,30
^ Fear of pain, anxiety about treatment duration, multiple radiographs, and uncertainty about outcomes are recurring reasons patients delay or avoid endodontic care.^20,24
^ Moreover, many patients still associate RCT with infection, failure, and unnecessary expense, and rely on home remedies or demand extraction to achieve rapid pain relief.

This problem is especially common in Saudi Arabia, where available studies indicate common oral health myths and misconceptions that may be influenced by educational and social factors. Previous Saudi studies from Riyadh and Taif have reported persistence of dental myths, knowledge gaps among adults, and the need for targeted educational interventions to improve oral health literacy and treatment outcomes.^1,4
^


Despite growing interest in dental misconceptions, endodontic myths remain insufficiently studied, particularly those related to the effects of RCT on human health. No population-based study has directly measured the prevalence of beliefs that RCT may cause cancer, chronic disease, or heart disease, nor has any study examined endodontic misconceptions’ association with preference for extraction rather than RCT. The present study contributes to the existing literature by quantifying the prevalence of 10 distinct endodontic myths and their associations with demographic characteristics and extraction preferences in a Saudi population. Therefore, the aim of this study was to assess the prevalence of endodontic misconceptions among dental patients in Madinah, Saudi Arabia, and to evaluate whether endorsement of these myths is associated with a preference for tooth extraction over RCT.

## Methods and Materials

This study is a cross-sectional observational study conducted among dental patients attending dental clinics in Almadinah Almunawarah, Saudi Arabia and was approved by the Research Ethics Committee of Taibah University (TU-DNT-26-02-04). A paper-based, self-administered questionnaire was distributed in person at 11 dental hospitals and private clinics in Almadinah Almunawarah, Saudi Arabia, from 10th of April to 8th of May 2026. Adults aged 18 and older who attended the dental clinics during the study period, without cognitive, hearing, or vision impairments, and were willing to complete the questionnaire were included. The questionnaire consisted of two sections: Section A gathered sociodemographic information, including age, gender, nationality, educational level, employment status, sources of health information, and whether they had previously undergone RCT. Section B comprised 10 self-administered closed-ended questions categorised into three domains: ‘Treatment Procedure Myths’, ‘Treatment Outcome Myths’, and ‘Systemic Health Myths’, and participants’ preferences for extraction versus RCT.

The questionnaire was developed following a comprehensive review of the published literature on prevalent dental misconceptions and patient knowledge regarding endodontic treatment in both global and Saudi contexts. Key studies were identified that highlighted specific myths related to oral health, endodontic treatments, and beliefs concerning systemic health and pregnancy.^1,7,16
^ The questionnaire was initially drafted in English and translated into Arabic to ensure clarity and cultural appropriateness for the target population. In addition, two experienced endodontists reviewed the questionnaire for content relevance and clarity. A pilot test of the questionnaire involved administering it to 15 participants at the University Dental Hospital clinics who were excluded from the main analysis, which confirmed the clarity of the questions and resulted in minor changes. The minimum sample size was calculated using the single-population proportion formula:


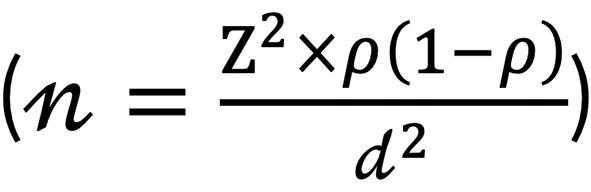
.

An expected true population proportion (*p*) of 50% was chosen to provide the most conservative estimate, and with a 95% confidence level (Z = 1.96) and a 5% margin of error (d = 0.05), the minimum required sample size was determined to be 385 participants. During the recruitment period, 570 dental patients were approached, of whom 463 completed the questionnaire and were included in the analysis, exceeding the minimum required size by approximately 20%. The remaining 107 individuals declined to participate, resulting in a response rate of 81.2%. Data were analysed using SPSS version 25.0 (IBM, Armonk, NY, USA) software program. Analysis included chi-square tests to examine relationships among demographics, myth endorsement, and extraction preference. Multivariable logistic regression was performed to determine whether individual myths remain independently associated with extraction preference after adjusting for age, gender, education and previous RCT. A *p* value below 0.05 was considered significant.

## Results

The questionnaire was filled out by 463 dental patients. The majority of participants were aged 18–30 years (53.1%), females (52.3%), and Saudi nationals (64.1%). About half (49.5%) had a university degree, 36.9% were employed, and 31.7% were students. Table 1 contains a complete demographic breakdown. More than two-thirds (70.6%) of the participants had previously undergone RCT. When asked about the source of their endodontic information, the majority of participants (55.9%) stated dentists, followed by social media and the internet (42.8%).

**Table 1 Table1:** Characteristics of the study participants (n = 463)

Variable	Category	n (%)
Age	18–30 years	246 (53.1)
	30–40 years	105 (22.7)
	40–50 years	69 (14.9)
	> 50 years	43 (9.3)
Gender	Male	221 (47.7)
	Female	242 (52.3)
Nationality	Saudi	297 (64.1)
	Non-Saudi	166 (35.9)
Education level	No formal education	5 (1.1)
	Primary	14 (3.0)
	Middle school	36 (7.8)
	High school	146 (31.5)
	University	229 (49.5)
	Postgraduate	33 (7.1)
Employment status	Employed	171 (36.9)
	Student	147 (31.7)
	Unemployed	117 (25.3)
	Retired	28 (6.0)
Source of dental information	Dentist	259 (55.9)
	Social media/Internet	198 (42.8)
	Family/Friends	95 (20.5)
	Personal experience	92 (19.9)
	Books	48 (10.4)
	TV/Radio	22 (4.8)
Previous root canal treatment	Yes	327 (70.6)
	No	136 (29.4)


Table 2 shows the extent to which endodontic myths were endorsed. The most widespread misconception was that root canal treatment must be done in multiple visits (73.0%), followed by the belief that antibiotics are necessary during the procedure (68.5%) and that RCT causes teeth to become weak and brittle (63.1%). More than a third of participants (38.0%) thought RCT should be avoided during pregnancy. Moreover, 30.9% believed that a root-canal-treated tooth remains infected and continues to harbour bacteria, and 19.7% believed that teeth that have undergone RCT should be extracted. Myths about systemic diseases were less common; these include beliefs that RCT causes cancer (6.3%), cardiovascular disease or heart attacks (10.8%), or chronic illnesses (14.3%) (Table 2).

**Table 2 Table2:** Prevalence of endodontic myth endorsement among study participants (n = 463)

No	Item	Prevalence of (Yes responses count)	(%)
Domain 1: Treatment procedure myths			
1	Do you believe that RCT cannot be completed in one visit and requires more than one visit?	338	73
2	Do you believe that an antibiotic should be taken when RCT is needed?	317	68.5
3	Do you believe that if there is pain in a particular tooth, placing a painkiller tablet on the tooth will relieve the pain?	169	36.5
4	Do you believe that if a pregnant woman has tooth pain and needs RCT, it should not be performed during pregnancy?	176	38
Domain 2: Treatment outcome myths			
5	Do you believe that after RCT, the tooth becomes weak and may break easily?	292	63.1
6	Do you believe that teeth that have undergone RCT are infected and a source of bacteria in the body?	143	30.9
7	Do you believe that teeth that have undergone RCT should be extracted?	91	19.7
Domain 3: Systemic health myths			
8	Do you believe that RCT can cause chronic diseases?	66	14.3
9	Do you believe that RCT can cause cardiovascular disease or heart attack/stroke?	50	10.8
10	Do you believe that RCT can cause cancer?	29	6.3


### Demographic Factors Associated with Endodontic Myth Endorsement

Several demographic factors were significantly associated with the extent to which participants endorsed endodontic myths (Table 3). Older participants (40 years and above) were more likely than younger participants (18–40 years) to believe that RCT should be completed over multiple visits (83.9% vs 69.5%, *p* = 0.021).

**Table 3 Table3:** Significant demographic factors associated with endodontic myth endorsement

Belief statement	Category of demographic factors		*p* value	Holm–Bonferroni-adjusted *p* value
	18–40 years old (n = 351)	40–50 and older (n = 112)		
RCT should be done in multiple visits	Yes: 244 (69.5%) No: 107 (30.5%)	Yes: 94 (83.9%) No: 18 (16.1%)	0.003	0.021
Belief statement	No formal / Primary / Middle school (n = 55)	High school/University/Postgraduate (n = 408)		
RCT causes cardiovascular diseases/ heart attacks	Yes: 14 (25.5%) No: 41 (74.5%)	Yes: 36 (8.8%) No: 372 (91.2%)	< 0.001	0.005
Painkiller tablet on tooth relieves pain	Yes: 31 (56.4%) No: 24 (43.6%)	Yes: 138 (33.8%) No: 270 (66.2%)	0.001	0.008
Belief statement	No prior RCT (n = 136)	Prior RCT (n = 327)		
Painkiller tablet on tooth relieves pain	Yes: 59 (43.4%) No: 77 (56.6%)	Yes: 110 (33.6%) No: 217 (66.4%)	< 0.001	0.005
Pearson’s chi-square test with the Holm–Bonferroni correction applied to adjust *p* values for multiple testing across the 10 endodontic myth variables (n = 463). Statistically significant values (*p* < 0.05).				

Education level was closely linked to belief in myths. Participants with less education (no formal education, primary, or middle school) were more likely to hold misconceptions than those with high school, university, or postgraduate education. For example, 25.5% of those with a lower education level believed that RCT causes cardiovascular diseases or heart attacks, compared to 8.8% of those with more education (*p* = 0.005). Similarly, more people with less education thought that putting a painkiller tablet directly on a tooth relieves pain (56.4% vs 33.8%, *p* = 0.008).

Previous experience with RCT was also associated with one endodontic myth. Participants who had not previously undergone RCT were more likely to believe that placing a painkiller tablet on a tooth relieves pain (43.4% vs 33.6%, *p* = 0.005) (Table 3).

### Association Between Endodontic Myths and Preference for Extraction

Most participants preferred RCT over tooth extraction (n = 322, 69.5%). However, significant associations were observed between endorsement of several endodontic myths and preference for tooth extraction over RCT (Table 4).

**Table 4 Table4:** Significant associations between endodontic myths and preference for extraction over root canal treatment

Endodontic myth	Endodontic myth endorsement	Prefer extraction n (%)	Do not prefer extraction n (%)	Unadjusted odds ratio (OR) [95% CI]	*p* value	Holm-adjusted *p* value
Painkiller on tooth relieves pain	yes	69/169 (40.8%)	100/169 (59.2%)	2.127 [1.418–3.191]	< 0.001	0.003
	no	72/294 (24.5%)	222/294 (75.5%)			
RCT causes cancer	yes	17/29 (58.6%)	12/29 (41.4%)	3.542 [1.644–7.629]	0.001	0.011
	no	124/434 (28.6%)	310/434 (71.4%)			
RCT-treated teeth remain infected	yes	57/143 (39.9%)	86/143 (60.1%)	1.862 [1.226–2.827]	0.005	0.033
	no	84/320 (26.3%)	236/320 (73.8%)			
RCT-treated teeth must be extracted	yes	118/202 (58.4%)	84/202 (41.6%)	7.929 [4.786–13.136]	< 0.001	< 0.001
	no	23/261 (8.8%)	238/261 (91.2%)			
Pearson’s chi-square test with the Holm–Bonferroni correction applied to adjust *p* values for multiple testing across the 10 endodontic myth variables (n = 463). Statistically significant values (*p* < 0.05).						

Belief that RCT causes cancer was associated with preference for extraction, with 58.6% of believers preferring extraction compared with 28.6% of non-believers (*p* = 0.011).

Similarly, participants endorsing the myth that RCT-treated teeth remain infected after treatment demonstrated a higher preference for extraction than those who did not hold this belief (39.9% vs 26.3%, *p* = 0.033). In addition, the belief that all root-canal-treated teeth should be extracted showed the strongest association with preference for extraction (58.4% vs 8.8%, *p* < 0.001).

Moreover, participants who believed that placing a painkiller tablet directly on a tooth relieves pain showed a higher preference for extraction compared with those who rejected this misconception (40.8% vs 24.5%, *p* = 0.003).

To assess the independent association between endodontic misconceptions and patient preference for tooth extraction over RCT, adjusted for possible confounders, a multivariable binary logistic regression was done. The model was adjusted for the participant’s age, gender, level of education, and history of RCT.

The belief that RCT-treated teeth must be extracted emerged as the strongest independent predictor of treatment preference (*p* < 0.001, aOR = 0.167, 95% CI [0.096, 0.292]). Participants holding this misconception were significantly less likely to favour RCT over extraction. Additionally, the belief that placing a painkiller tablet directly on a tooth reduces pain showed a statistically significant association with preference (*p* = 0.006, aOR = 0.517, 95% CI [0.324, 0.825]).

All other evaluated myths and adjustment covariates showed no statistically significant independent effect in the multivariable model (*p* > 0.05) (Table 5).

**Table 5 Table5:** Multivariable binary logistic regression predicting preference for tooth extraction

Variable	B	SE	Wald	df	*p* value	Adjusted odds ratio (aOR)	95% CI for Exp(B)
Adjustment covariates							
Gender	−0.388	0.234	2.751	1	0.097	0.678	0.428–1.073
Education level	0.221	0.365	0.369	1	0.544	1.248	0.611–2.550
Age	0.329	0.278	1.398	1	0.237	1.389	0.806–2.394
Previous RCT	0.222	0.256	0.753	1	0.386	1.249	0.756–2.065
Endodontic myths & misconceptions							
Myth 1: Multiple visits are a must	0.215	0.269	0.636	1	0.425	1.239	0.732–2.099
Myth 2: Antibiotics are always needed	−0.43	0.264	2.644	1	0.104	0.651	0.388–1.092
Myth 3: Painkiller on tooth relief pain	−0.66	0.238	7.666	1	0.006	0.517	0.324–0.825
Myth 4: No RCT during pregnancy	0.362	0.244	2.198	1	0.138	1.436	0.890–2.318
Myth 5: Teeth are brittle after RCT	0.151	0.242	0.389	1	0.533	1.163	0.724–1.868
Myth 6: Source of bacteria in body	−0.176	0.269	0.429	1	0.512	0.838	0.495–1.420
Myth 7: RCT-treated teeth must be extracted	−1.79	0.285	39.546	1	< 0.001	0.167	0.096–0.292
Myth 8: Cause chronic illness	−0.053	0.367	0.021	1	0.885	0.948	0.462–1.948
Myth 9: Cause heart attack/stroke	−0.226	0.39	0.335	1	0.563	0.798	0.371–1.714
Myth 10: Cause cancer	−0.521	0.488	1.14	1	0.286	0.594	0.228–0.545
Constant	1.32	0.493	7.184	1	0.007	3.744	


## Discussion

This study found that misconceptions about RCT are highly prevalent in the studied population, and that several specific beliefs were significantly associated with a preference for extraction over endodontic therapy.

### Prevalence of Endodontic Myths

When looking at patients’ dental information sources, dentists and doctors were the most common sources of endodontic information (55.9%), followed by social media and the internet (42.8%). Although professional advice constitutes the most popular source, the fact that a majority relies on social media sites is rather alarming. In their analysis of online oral health information, Lotto et al (2023) found that 11.6% of all online misinformation involved RCT, with toxicity issues among the common themes.^19^ Recent research into root canal misinformation posted on social media found that stories often contained not only misinformation but also sensationalism, with some posts even created by dentists.^16^


The most widespread misbelief is that RCT must happen over several visits and cannot be completed in a single session. This is consistent with findings from Alamri and Aldawsari (2017), who found that misconceptions about the number of visits required were common in a Saudi population and reported that only 20.9% believed that the RCT could be done in a single visit.^2^ The persistence of this myth may reflect earlier endodontic teaching that emphasised multi-visit protocols; however, contemporary evidence supports single-visit treatment as a safe and effective alternative in appropriately selected cases.^21,22
^


A majority of the participants believed that antibiotics are required when RCT is required (68.5%) and that placing a painkiller tablet directly on a painful tooth relieves pain (36.5%). These beliefs are consistent with the reported pattern of dental antibiotic misuse and self-medication in Saudi Arabia. Antibiotics were prescribed for nearly 30% of more than 43,000 dental visits in the Albaha region, and more than a quarter were unwarranted.^8^ Moreover, patient pressure seems to be part of this issue; Al-Khatib and Al-Mohammad (2022) found that 33.9% of Saudi dentists received weekly requests for unnecessary antibiotics, and that such demands affect prescribing behaviour.^5^


An overwhelming majority of respondents (63.1%) believed that teeth become weak and brittle after root canal therapy. From a biomechanical viewpoint, the public might be led to believe that dehydration or perhaps the loss of the internal neurovascular supply is responsible for this; however, modern evidence in endodontics shows that moisture loss following RCT causes almost no change in the hardness or elasticity of dentine.^14^ Fracture susceptibility is mainly induced by structural loss during cavity access preparation. Therefore, it is essential to follow a conservative approach clinically to enhance survival. By employing conservative techniques in the preparation of endodontically treated teeth, we can ensure better long-term fracture resistance as well as greater structural integrity.^26^


The belief in applying topical analgesics to the tooth surface is in line with the region’s prevalence of oral health self-medication practices. This is consistent with the study by Abdul Salam TA et al. (2023), which found that 34.4% of participants believed ‘Placing a (painkiller) tablet on a tooth reduces pain’.^1^ Assiri et al (2021) reported that 40% of participants in the Aseer region of Saudi Arabia used household ingredients such as apple vinegar, turmeric, lemon, and baking soda for oral care, with the majority learning these practices from family members. Such unorthodox home remedies may delay professional care and lead to avoidable complications.^10^


### Demographic Associations

Older participants endorsed having RCT in multiple visits more than younger participants. This is consistent with findings from a recent Saudi study on dental misconceptions.^4^ Older adults may have been exposed to earlier dental practices and information settings, and they may have less access to contemporary health information.

Education level was found to be an important factor, with lower education levels associated with a higher likelihood of believing in endodontic myths. One of the most consistent findings in health literacy research is that lower educational attainment is associated with greater endorsement of health misconceptions, and our data confirm this pattern in an endodontic context. King et al (2023) found that low oral health literacy was associated with poor knowledge about causes and consequences of oral disease and reduced agency in managing oral health.^17^ Similarly, education has been found to be a strong predictor of health knowledge across several conditions in multiple Saudi studies.^3,6
^


Participants who had not previously undergone RCT were more likely to believe pain can be resolved by placing a painkiller tablet on the tooth, which is understandable since they lack a personal, first-hand experience of the procedure. This highlights the potential role for patient education during and after treatment as a tool for countering misconceptions.

### Tooth Extraction Preference Associations

A considerable proportion of the study participants believed that root-canal-treated teeth remain infected and a source of bacteria in the body, and such perceptions showed significant correlation with a preference for extraction over RCT. This misunderstanding probably originates from the focal infection concept, which existed between 1930 and 1950, leading to the routine extraction of infected teeth.^13^ While the concept has greatly evolved since then, current scientific evidence has established intricate reciprocal connections between oral infections and systemic disorders. For instance, periodontal infection has links with cardiovascular disease and diabetes, but the evidence specific to endodontic infections is weaker, and causality remains unclear.^27,28
^ A recent interdisciplinary consensus in the Czech Republic affirmed that the oral-systemic axis is more complex than the classical focal infection theory suggests, and that routine extraction of root-filled teeth is no longer justified.^23^ In the present study, beliefs related to the fear that RCT can cause cancer were significantly associated with extraction preference. Shetty et al (2024) studied a sample of rural Indians and found that 60.2% preferred extraction over RCT. Cost and fear were the most prominent drivers.^29^ Thus, these observations were made in a Saudi context.

The analyses in the present study showed that preference for tooth extraction over RCT was attributable predominantly to systemic health concerns and beliefs about cancer and the persistence of the infection. By contrast, the procedural aspects of tooth fragility, multiple visits, and treatment restrictions during pregnancy did not show significant association. This pattern suggests that fears about infection and health impact primarily influence patient preference for extraction over RCT. According to the American Association of Endodontists, myths linking RCT to cancer and systemic illness stem from century-old, scientifically flawed theories that have been thoroughly refuted by modern medical and dental evidence.^9^


Furthermore, previous literature shows that poor knowledge about endodontic outcomes and fear of treatment failure represent significant barriers to acceptance of root canal therapy.^11,21
^ As a result, the dentist should conduct evidence-based communication to bridge the gap between patient perception and clinical reality to preserve natural dentition.

### Limitations

This study has limitations; these include the use of self-reported data and the cross-sectional nature of the study. The multicentre design provided a better test of the research hypothesis than a single-centre design, but the specific cultural context of Madinah city may also affect generalisability. Another limitation of this study is that participants were recruited exclusively from dental clinics, which may limit the generalisability of the findings to the general population; future studies should use community-based, multicentre sampling. Although the study questionnaire was designed following a comprehensive review of the published literature, underwent pilot testing, and was reviewed by two experienced endodontists, it was not psychometrically validated. This includes structured content validity, face validity, construct validity, internal consistency, and test-retest reliability. This limitation was partly related to the exploratory design of the study, as the questionnaire evaluated several distinct endodontic misconceptions instead of a single underlying construct. The results should be interpreted as item-level associations rather than as scores from a fully validated instrument. Moreover, future investigations should employ longitudinal designs, recruit broader samples, and examine variations across different geographic regions.

## Conclusion

Within the limitations of this survey-based study, endodontic myths are widespread among patients in Almadinah Almunawara. Certain myths, specifically systemic health concerns, misconceptions regarding infection persistence, and fears linking root canal therapy to cancer, are strongly associated with a preference for tooth extraction over RCT. Specific demographic factors such as age, educational level, and previous RCT experience influence the endorsement of particular misconceptions. Addressing these misconceptions through targeted patient education and enhanced clinical communication is essential to dispel historical myths, improve treatment acceptance, and promote natural dentition retention.

### Acknowledgements

The authors thank the patients who participated in this study for their time and cooperation. We also acknowledge the dental clinics in Almadinah Almunawarah that facilitated the study.

## References
